# Epidemic spreading on complex networks with community structures

**DOI:** 10.1038/srep29748

**Published:** 2016-07-21

**Authors:** Clara Stegehuis, Remco van der Hofstad, Johan S. H. van Leeuwaarden

**Affiliations:** 1 Eindhoven University of Technology, Department of Mathematics and Computer Science, P.O. Box 513, 5600 MB Eindhoven, The Netherlands

## Abstract

Many real-world networks display a community structure. We study two random graph models that create a network with similar community structure as a given network. One model preserves the exact community structure of the original network, while the other model only preserves the set of communities and the vertex degrees. These models show that community structure is an important determinant of the behavior of percolation processes on networks, such as information diffusion or virus spreading: the community structure can both *enforce* as well as *inhibit* diffusion processes. Our models further show that it is the mesoscopic set of communities that matters. The exact internal structures of communities barely influence the behavior of percolation processes across networks. This insensitivity is likely due to the relative denseness of the communities.

Many complex systems across the sciences can be modeled as networks of vertices joined in pairs by edges. Examples include the internet and the world-wide web, biological networks, food webs, the brain, neural networks, communication and transport networks, and social networks. This has spurred a tremendous interest in developing mathematical models that can capture universal network properties. Moreover, with network data describing network topologies, properties derived from models can be tested against real-world networks.

The behavior of dynamic processes such as percolation or epidemic models on those networks are of significant interest, since for example they model the spreading of information or a virus across a network^1^,^2^,^3^,^4^. Understanding models for percolation may enhance insight in how an epidemic can be stopped by immunization, or how a message can go viral by choosing the right initial infectives. An important question is how the structure of the network affects the dynamics of the epidemic^5^. A vast amount of research focuses on scale-free networks that possess a power-law degree distribution^6^,^7^,^8^,^9^,^10^, so that the probability *p*_*k*_ that a vertex has *k* neighbors scales with *k* as *p*_*k*_ ~ *ck*^−*τ*^ for some constant *c* and characteristic exponent *τ* > 1. The power-law distribution leads to scale-free behavior such as short distances due to the likely presence of *hubs* or high-degree vertices. The characteristic exponent *τ* was also found to play a central role in various percolation processes^11^,^12^,^13^,^14^,^15^. Other authors have focused on the influence of clustering on the spread of epidemics^16^,^17^,^18^,^19^,^20^.

Real-world networks, however, are not completely characterized by their microscopic and macroscopic properties. Many real-world networks display a community structure^21^, where groups of vertices are densely connected, while edges between different groups are more scarce. Since communities are small compared to the entire network, but seem to scale with the network size, they are typically of mesoscopic scale^22^,^23^. The problem of detecting the community structure of a network has received a lot of attention^22^,^24^. The exact way in which communities influence the properties of a network is a different problem. For example, the community structure of a network influences the way a cooperation process behaves on real-world networks^25^, and using community structure improves the prediction of which messages will go viral across a network^26^. Several stylized random graph models with a community structure have shown that communities influence the process of an epidemic across a network^27^,^28^,^29^,^30^,^31^,^32^,^33^,^34^, but the extent to which community structure affects epidemics on real-world networks is largely unexplained. Our main goal is to enhance our understanding of the intricate relation between community structures and the spread of epidemics, and in particular to identify the properties of community structures that have the largest influence.

We study two random graph models that generate networks with a similar community structure as any given network. We find that these models capture the behavior of epidemics or percolation on real-world networks accurately, and that the mesoscopic community structure is vital for understanding epidemic spreading. We find that the sets of communities are of crucial importance, while quite surprisingly, the precise structure of the intra-community connections hardly influences the percolation process. Furthermore, we find that community structure can both enforce as well as inhibit percolation.

## Models

**Figure 1:**
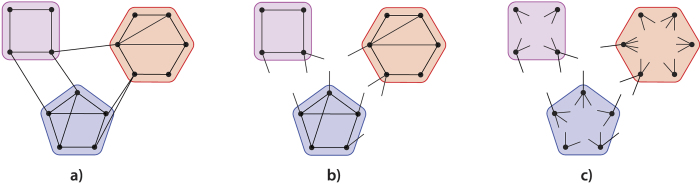
HCM and HCM* illustrated. (**a**) A network with 3 communities. (**b**) HCM randomizes the edges between different communities. (**c**) HCM* also randomizes the edges inside the communities.

We now introduce two random graph models in detail. For a given real-world network, both models randomize the edges of the network, while keeping large parts of the community imprint. Suppose that we are given the set of communities of a particular real-world network. Then the first model, the hierarchical configuration model (HCM), keeps all edges inside the communities^35^,^36^, while rewiring the inter-community edges. Indeed, all inter-community edges are replaced by two half-edges, one at each end of an inter-community edge. Then, one by one, these half-edges are paired at random. Thus, in HCM, the precise community structure of the network is the same as in the original network, but the inter-community connections are random. The second model (HCM*), introduced as the modular random graph in ref. 37, replaces both the inter-community edges and the intra-community edges by pairs of half-edges. Then again, the half-edges are paired at random. An additional constraint is that all inter-community half-edges must be paired to one another, and all half-edges corresponding to the same community must be paired to one another (see Fig. 1 and Supplementary Note 3). Thus, a network generated by HCM* is completely random, except for the set of communities and the degree distributions inside and outside the communities.

HCM and HCM* are extensions of the configuration model (CM), a random graph with a given degree distribution. The CM has received enormous attention in the network literature, due to the combination of its simplicity and its flexibility in choosing an appropriate degree structure^38^,^39^. CM only preserves the microscopic degree distribution of the real-world networks, while HCM* also preserves the mesoscopic community structure. HCM instead, preserves the entire community structure. Supplementary Table 3 shows that indeed most of the community structures of the original networks and the networks generated by HCM and HCM* are similar. Therefore, if we sort the random graph models in decreasing randomness, we first have CM, then HCM*, and then HCM. When comparing the behavior of an epidemic process on these random graphs to the original network, we see how much of the behavior of epidemics on real-world networks can be explained by its degree distribution (CM), its rough community structure (HCM*), and by the exact community shapes (HCM). The aim of this paper is to investigate to which extent microscopic and mesoscopic network properties determine the spread of epidemics.

The fixed community shapes combined with the randomized inter-community connections make HCM analytically tractable^35^. However, keeping all intra-community edges fixed makes HCM prone to overfitting. HCM* does not have this problem and is more suitable to generate a random network with a community structure, since all edges within communities are randomized. Randomizing the intra-community edges makes HCM* harder to analyze analytically than HCM. Some analytical results of HCM, however, can be extended to results of HCM* (Supplementary Note 3).

## Results

We analyze six different real-world networks: the internet on the Autonomous Systems level^40^, an email network of the company Enron^40^,^41^, the PGP web of trust^42^, a collaboration network in High energy physics, extracted from the arXiv^40^, a Facebook friendship network^43^ and an interaction network between proteins in yeast^44^. Table 1 shows several statistics of these data sets and their community structures. We extract the communities of these networks with the Infomap community detection algorithm^45^, and use these communities as input for the HCM and HCM* model, to create networks with a similar community structure as the original networks. Table 1 shows that the communities are of mesoscopic size: while the communities are small compared to the entire network, and have a small expected size, all networks still contain a few large communities.

An important property of a network is its connectedness, expressed by the fraction of vertices in the largest component. For HCM, the size of the largest component can be derived analytically (Supplementary Note 3). This size is independent of the precise community shapes, and therefore is the same for HCM and HCM*, as long as the communities of HCM* remain connected. Supplementary Note 3.3 shows that most HCM* communities indeed remain connected. The size of the largest component of real-world networks can be well predicted using the analytical estimates of HCM, which only uses the joint distribution of community sizes and the number of edges going out of the communities (Table 2). These estimates yield a considerable improvement compared to CM, which is generally a few percent off.

**Figure 2:**
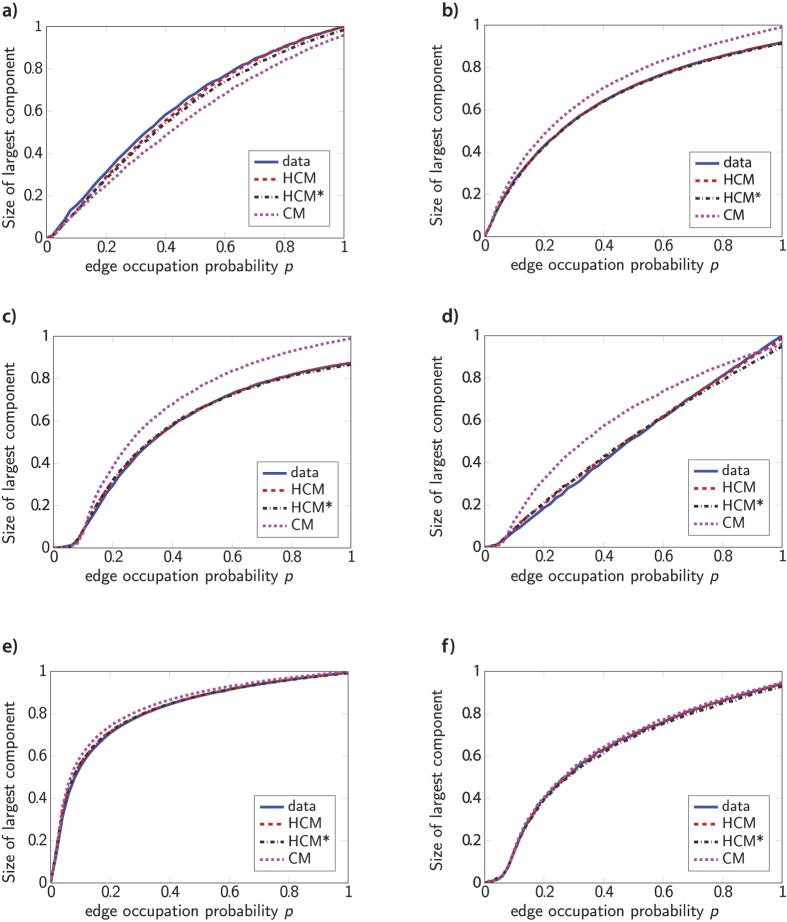
HCM, HCM* and CM under bond percolation compared to real-world networks. (**a**) Autonomous Systems network (**b**) Enron email network (**c**) Collaboration network in High energy physics (**d**) PGP network (**e**) Facebook friendship network (**f**) yeast network. Independently, each edge is deleted with probability 1 − *p*. The size of the largest component after deleting the edges is the average of 500 generated graphs.

The long-term properties of an epidemic outbreak can be mapped into a suitable bond percolation problem. In this framework, the probability *p* that a link exists is related to the probability of transmission of the disease from an infected vertex to a connected susceptible vertex. The latter corresponds to removing edges in a network with probability 1 − *p* and keeping the edges with probability *p* independently across edges (other types of epidemics are discussed in Supplementary Note 4). A quantity of interest is the size of the largest component as a function of *p*, which can be described analytically for HCM^35^. However, this size depends on the community shapes, and therefore bond percolation on HCM does not necessarily give the same results as percolation on HCM*. Inspired by the insensitivity of the giant component to the exact community shapes, we establish whether the community shapes significantly influence the size of the giant percolating cluster by simulation, by showing how bond percolation affects the connectivity of the original networks, compared to CM, HCM and HCM* (Fig. 2).

We see that the behavior of the real-world networks under bond percolation is captured accurately by both HCM and HCM*, in contrast to CM. In Supplementary Figs 1–5, we see that HCM and HCM* also perform well for other types of percolation processes and an SIR epidemic. These results reveal and confirm the key role of the mesoscopic community structure in percolation processes. Furthermore, the fact that the predictions of HCM and HCM* are *both* close to the behavior of the original network under percolation indicates that the shapes of the communities only have minor influence on the percolation process. The surprising finding that the exact internal community structure barely influences the epidemic processes may be explained by the denseness of the communities. Table 1 shows that the communities are very dense compared to the entire network. Since community detection algorithms look for dense subsets in large complex networks, applying HCM or HCM* to real-world networks typically yields sets of dense communities. The Autonomous Systems network has communities that are much less dense than in most other networks^46^, but even in that network the communities are much denser than the entire network. Therefore, in the case of bond percolation for example, the communities of mesoscopic size are supercritical, and the communities will be almost connected after percolation. Thus, an epidemic entering a community of mesoscopic size will reach most other community members. It is more difficult for the epidemic to reach other communities, which makes the inter-community edges the important factor for the spread of an epidemic. When generating a HCM* network, the communities stay of the same denseness, and therefore it is still relatively easy for the epidemic to spread inside the communities, regardless of their exact shapes.

The only process where HCM and HCM* are not always close to the process on the original graph, is a targeted attack (Supplementary Fig. 2), even though both models still outperform CM. Furthermore, some networks show a difference between the predictions of HCM and HCM*. Therefore, the exact community structures may have some influence on a targeted attack on a real-world network. Another interesting observation is that where most networks are highly sensitive to a targeted attack, the Facebook network has a community structure that makes it more resistant against a targeted attack than a configuration model. This particular feature of the Facebook network can be explained by the fact that in the Facebook network, most vertices of high degree are in the same community. Therefore, deleting high degree vertices has a smaller effect than in a corresponding CM model.

The results of the yeast network show that in some situations CM performs equally well as HCM or HCM*. Thus, in some cases the mesoscopic properties of a network do not influence percolation processes. In the case of the yeast network, this can be explained by its almost tree-like structure; there is no noticeable community structure. Thus, by adding the community structure in HCM or HCM*, no structural information is added. This suggests that CM, HCM and HCM* combined can also show whether the community structure given by a community detection algorithm is meaningful. When the behavior of various processes on CM, HCM and HCM* are similar, this may imply that there is no real community structure in the network.

The ENRON, High energy physics and PGP networks have communities that inhibit percolation or an SIR epidemic compared to a configuration model with the same degree distribution. This is similar to the observation that communities can act as traps for an epidemic process across a network^47^. In contrast, the communities in the Autonomous Systems graph enforce the percolation process, which may be attributed to its star-like community structure. Since HCM* preserves the degrees of the vertices inside their own community, HCM* creates a graph that captures this star-like structure. An important conclusion is that these findings confirm that both HCM and HCM* are realistic models for real-world networks.

Where ref. 48 creates a reshuffling of a given network using several microscopic properties of every vertex, HCM and HCM* use mesoscopic properties instead. An advantage of using HCM or HCM* is that both models are easy to generate. Since HCM* is more random than HCM, it is a better choice for generating a random network. Note that in HCM*, the rewiring of intra-community edges makes the community structure a uniform simple graph with the prescribed degrees. Specifically, if the interest is to generate a random graph such that percolation on that graph behaves in a similar way as in the original network, then our results show that HCM* is a suitable choice. However, HCM* does not capture the microscopic properties of the original network as effectively as HCM. HCM*, for example, does not generate networks with similar clustering as in the original network^37^. Therefore, when the goal is to create a network with similar clustering as the original network, using HCM* may be less suitable. Indeed Table 3 shows that in most cases HCM generates a network with a clustering coefficient that is closer to the value of the original network. An exception is the Autonomous Systems network, where HCM* is closer to the real value of the clustering. An explanation for this is that the communities in the Autonomous Systems network have virtually no clustering; all clustering is between different communities. HCM also has no clustering inside the communities, but the pairing between different communities destroys the clustering between different communities, and therefore HCM creates a network with a lower clustering coefficient. HCM* also destroys the clustering between different communities, but by rewiring the edges inside communities, creates some clustering inside the communities. Therefore, the value of the clustering of HCM* is closer to the value of the original network than the one of HCM.

The fact that HCM* does not capture the clustering coefficient and the assortativity (See Supplementary Notes 3.1) well, but does capture the spread of an epidemic across a network, again confirms that the mesoscopic properties are of vital importance for the spread of an epidemic across a network. Even though microscopic features such as clustering are destroyed in HCM*, the mesoscopic properties are sufficient to know how an epidemic spreads, making HCM* a suitable random graph model when considering the mesoscopic structure of networks.

## Conclusion

Community structures in real-world networks have a profound impact on percolation or epidemic spreading, which is central to our understanding of dynamical processes in complex networks. The theoretical analysis of epidemic spreading in heterogeneous networks with community structure requires the development of novel analytical frameworks. We have introduced the hierarchical configuration model (HCM) to describe such networks. Both HCM and its randomized counterpart HCM* turn out be highly suitable to capture epidemic spreading on real-world networks. We have shown this by mapping the models to various real-world networks, and by investigating a range of epidemic processes including bond percolation, bootstrap percolation and an SIR epidemic. Our experiments show that while it is essential to take the community structure into account, the precise internal structure of communities is far less important for describing an epidemic outbreak. This insensitivity is likely due to the relative denseness of the communities. When communities are sparse, their internal structures are expected to have a more decisive effect on epidemic spreading. The HCM and HCM* models can easily be extended to include overlapping communities, by considering an auxiliary graph. It would be interesting to see whether including overlapping communities further improves the description of percolation across complex networks.

## Additional Information

**How to cite this article**: Stegehuis, C. *et al.* Epidemic spreading on complex networks with community structures. *Sci. Rep.* **6**, 29748; doi: 10.1038/srep29748 (2016).

## Supplementary Material

Supplementary Information

## Figures and Tables

**Table 1:** Statistics of the data sets.

	*N*	〈*s*〉	*s*_max_	*δ*_netw_	*δ*_com_	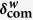
**AS**	11,174	21	910	3.75 · 10^−4^	0.38	0.10
**Enron**	36,692	15	1,722	2.73 · 10^−4^	0.73	0.22
**HEP**	9,877	10	181	5.33 · 10^−4^	0.59	0.32
**PGP**	10,680	12	160	4.26 · 10^−4^	0.41	0.24
**FB**	63,731	29	2,247	4.02 · 10^−4^	0.41	0.14
**yeast**	2,361	9	97	2.57 · 10^−3^	0.55	0.25

*N* is the number of vertices in the network, 〈*s*〉 the average community size, *s*_max_ the maximal community size. The denseness of the network *δ*_netw_ is defined as the number of edges divided by the number of edges in a complete graph of the same size. *δ*_com_ equals the average denseness of the communities, and 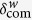 the average denseness of the communities weighted by their sizes (See Supplementary Note 1 for more information about these statistics).

**Table 2:** The size *S* of the giant component in the data sets compared to the analytical estimates of HCM and CM.

	*S* (data)	*S* (HCM)	*S* (HCM*)	*S* (CM)
**AS**	1.000	1.000	1.000	0.960
**Enron**	0.918	0.918	0.918	0.990
**HEP**	0.875	0.875	0.875	0.990
**PGP**	1.000	1.000	1.000	0.960
**FB**	0.995	0.995	0.995	0.999
**yeast**	0.941	0.941	0.941	0.948

**Table 3:** Average clustering for the original data set, HCM, HCM* and CM.

	Data	HCM	HCM*	CM
**AS**	0.30	0.16	0.20	0.09
**Enron**	0.50	0.35	0.22	0.03
**HEP**	0.47	0.40	0.24	0.00
**PGP**	0.26	0.24	0.19	0.00
**FB**	0.22	0.15	0.08	0.00
**yeast**	0.13	0.12	0.12	0.01

The presented values are averages of 100 generated graphs.
