# Supplementary material for: Epidemic spreading on complex networks with community structures
Source: Sci Rep. 2016 Jul 21;6:29748. doi: 10.1038/srep29748 (PMC4954979; doi:10.1038/srep29748)
Supplement: Supplementary Information [file srep29748-s1.pdf]

Supplementary material to “Epidemic spreading on complex networks with community structures”

Clara Stegehuis, Remco van der Hofstad, Johan S. H. van Leeuwaarden

Supplementary Notes

Supplementary Note 1 Network properties

Tables ??, ?? and ?? give several statistics of the networks that we have considered. We now explain these characteristics in more detail.

1.1 Graphs

A graph $G = (V, E)$ consists of a set of vertices V, and a set of edges E. In this paper, all graphs are undirected. The edge set E consists of pairs of vertices that are linked to one another, so that if $\{v_1, v_2\} \in E$, then v_1 and v_2 have an edge between them. The degree of vertex i is denoted by d_i, and represents the number of edges that are adjacent to vertex i. Let N denote the number of vertices in the graph, and N_k the number of vertices in the graph of degree k. Then the degree distribution of the graph is given by $p_k = N_k/N$, for $k = 0, 1, \dots$.

1.2 Clustering

The clustering coefficient C_i of a vertex i is defined as the number of triangles that i is part of, T_i, divided by the number of pairs of neighbors of vertex i, so that

$$C_i = \frac{2T_i}{d_i(d_i - 1)}. \tag{1}$$

This can be interpreted as the fraction of neighbors of v that are also neighbors of one another. Given the degree d_i of vertex i, the number of pairs of neighbors of i equals $d_i(d_i - 1)/2$. Then the average clustering coefficient C is defined as the average of the clustering coefficient of all vertices,

$$C = \frac{1}{N} \sum_{i=1}^{N} C_i. \tag{2}$$

1.3 Modularity

The modularity of a network is a measure of how well the network can be divided into communities. Consider a partition P of the N vertices into communities. The modularity $M(P)$ of a partition P equals [49]

$$M(P) = \sum_{c \in P} \frac{L_c}{L} - \left(\frac{d_c}{2L}\right)^2, \tag{3}$$

where L is the number of edges of the network, L_c is the number of edges inside community c, and d_c is the sum of all degrees of vertices in community c. This is a measure of how many edges are inside the community, minus how many edges would be expected if the vertices were connected at random. Therefore, a higher modularity implies many edges inside communities.

1.4 Assortativity

The assortativity of a graph $G = (V, E)$ can be interpreted as the correlation between the degrees at the end of a randomly chosen edge [50] and is given by

$$r(G) = \frac{2\sum_{\{i,j\} \in E} d_i d_j - \frac{1}{2L}\left(\sum_i d_i^2\right)^2}{\sum_i d_i^3 - \frac{1}{2L}\left(\sum_i d_i^2\right)^2}. \tag{4}$$

Positive assortativity indicates that vertices of high degree are connected to other vertices of high degree, and negative assortativity indicates that high degree vertices are typically connected to vertices of low degree. Assortativity is a frequently used network statistic, despite its dependence on the network size [51].

1.5 Size of giant component

A network may consist of several connected components, $\mathscr{C}_1, \mathscr{C}_2, \ldots, \mathscr{C}_k$. The proportion of vertices in the giant component S is then defined as the fraction of vertices in the largest connected component: $S = |\mathscr{C}_{\max}|/N$, where $\mathscr{C}_{\max}$ is the largest connected component in the network, i.e., $|\mathscr{C}_{\max}| = \max_i |\mathscr{C}_i|$, where $|\mathscr{C}|$ denotes the size of cluster $\mathscr{C}$. If the maximal component is not unique, we break ties in an arbitrary way.

1.6 Graph distances

The graph distance between two vertices u and v is defined as the minimal number of edges in a path that links u and v. Supplementary Figure 7 presents the graph distances for the different data sets. In some instances, HCM and HCM* capture the graph distances better than CM. However, for example for the yeast network, the distances in CM are already close to the distances in the original data set.

Supplementary Note 2 Community detection

The HCM and HCM* models use as input the community structure of a network. Several algorithms to detect this community structure are available. In this paper, we used the Infomap community detection algorithm [45]. This community detection algorithm uses a random walk perspective to detect the communities. It has a computational complexity of $O(N\log(N))$ for a network of N vertices, making it applicable to detect community structures in large networks. Furthermore, the algorithm performs well on several benchmarks compared to other community detection methods [52].

Supplementary Note 3 HCM and HCM*

Given a real-world network and the collection of its communities, obtained e.g., using a community detection algorithm, we construct HCM and HCM* in the following way. First, rewire the edges between different communities, using the switching algorithm. Select two inter-community edges uniformly at random, $\{u,v\}$ and $\{w,x\}$. Now delete these edges and replace them by $\{u,x\}$, $\{w,v\}$ if this results in a simple graph. Otherwise keep the original edges $\{u,v\}$ and $\{w,x\}$. This randomizes the inter-community edges uniformly if this procedure is repeated at least $100E$ times, where E is the number of inter-community edges [53]. This creates HCM.

To create HCM*, the edges within the communities are also randomized after rewiring the inter-community edges, again using the switching algorithm. This is repeated for all communities.

Now we analyze HCM in more detail, to analytically derive the size of its largest component as in [35]. Let s_i be the size of community i, and k_i the number of half-edges from community i to other communities. We call k_i the *inter-community degree* of community i. We define the joint distribution $p_{k,s}$ to be the fraction of communities of size s with inter-community degree k. We define two distributions and their probability generating functions to calculate the size of the largest component. The *excess inter-community degree distribution*

$$q_{k,s} = \frac{(k+1)p_{k+1,s}}{\langle k\rangle}, \tag{5}$$

can be interpreted as the probability to arrive in a community with inter-community degree k and size s when traversing a random inter-community edge, excluding the traversed edge. Here $\langle k\rangle = \sum_{k,s} kp_{k,s}$ is the expected value of k. Similarly, define

$$r_{k,s} = \frac{sp_{k,s}}{\langle s\rangle} \tag{6}$$

as the probability that a randomly chosen vertex is in a community of size s (including the vertex itself) and has k edges to other communities. The probability generating functions of these distributions are given by

$$g_q(x) = \sum_{k,s} q_{k,s} x^k = \frac{1}{\langle k \rangle} \sum_{k,s} k p_{k,s} x^{k-1}, \tag{7}$$

$$g_r(x) = \sum_{k,s} r_{k,s} x^k = \frac{1}{\langle s \rangle} \sum_{k,s} s p_{k,s} x^k, \tag{8}$$

and are used to calculate the asymptotic size of the largest component.

Let u be the probability that a community that is reached by traversing a random inter-community edge is not in the giant component, in which case all the communities connected to it cannot be in the giant component either. The k neighboring communities of the reached community are not in the giant component with probability u^k. Hence, a community is not in the giant component with probability

$$u = \sum_{k,s} q_{k,s} u^k = g_q(u). \tag{9}$$

The probability that a randomly chosen vertex is not in the giant component is $\sum_{k,s} r_{k,s} u^k = g_r(u)$. Thus, the proportion of vertices in the largest component S satisfies

$$S = 1 - g_r(u). \tag{10}$$

Equations (9)-(10) can be solved together to find the asymptotic size of the largest component.

Equations (9)-(10) only depend on the community sizes and the number of edges to other communities. Therefore, when the communities of HCM* are connected, they give the size of the largest component both for HCM and HCM*. In most instances, the number of edges from one vertex of the community to other vertices of the community is large, so HCM* typically generates connected communities, and indeed (10) can also be used to calculate the size of the giant component in HCM*.

3.1 Assortativity of HCM

The assortativity of the HCM can be computed analytically using (4). We denote the degree of a randomly chosen vertex among the N vertices of the graph by D_N. Then, (4) can be rewritten as

$$\begin{aligned} r(G) &= \frac{2\sum_{\{i,j\}\in E} d_i d_j - \frac{1}{2L}\left(\sum_i d_i^2\right)^2}{\sum_i d_i^3 - \frac{1}{2L}\left(\sum_i d_i^2\right)^2} = \frac{\frac{2}{N}\sum_{\{i,j\}\in E} d_i d_j - \frac{\left(\frac{1}{N}\sum_i d_i^2\right)^2}{\frac{1}{N}\sum_i d_i}}{\frac{1}{N}\sum_i d_i^3 - \frac{\left(\frac{1}{N}\sum_i d_i^2\right)^2}{\frac{1}{N}\sum_i d_i}} \\ &= \frac{\frac{2}{N}\sum_{\{i,j\}\in E} d_i d_j - \frac{\mathbb{E}[D_N^2]^2}{\mathbb{E}[D_N]}}{\mathbb{E}[D_N^3] - \frac{\mathbb{E}[D_N^2]^2}{\mathbb{E}[D_N]}}. \end{aligned} \tag{11}$$

Therefore, the only term of assortativity that depends on the community structure of HCM is the first term in the numerator. The edges of HCM can be split into two sets: the edges that are entirely inside a community, and the edges that are between two different communities, denoted by E_c and E_b respectively. The edges inside communities are fixed given the community shape. Let the n communities of the network be denoted by $\{H_1, \dots, H_n\}$. For a given community H, let $Q(H)$ denote

$$Q(H) = \sum_{\{i,j\}\in E_H} d_i d_j. \tag{12}$$

Then the contribution of the intra-community edges to the first term in the numerator can be written as

$$\mathbb{E}\Big[\frac{1}{N}\sum_{\{i,j\}\in E_c} d_i d_j\Big] = \frac{1}{N}\sum_{k=1}^{n}\sum_{\{i,j\}\in E_{H_k}} d_i d_j = \frac{1}{n\mathbb{E}[S_n]}\sum_{k=1}^{n} Q(H_k) = \frac{\mathbb{E}[Q_n]}{\mathbb{E}[S_n]}, \tag{13}$$

where $\mathbb{E}[Q_n]$ is the expected value of Q of a randomly chosen community, and $\mathbb{E}[S_n]$ the size of a uniformly chosen community. Let $D_N^{(b)}$ denote the number of edges to other communities of a randomly chosen vertex, and $L^{(b)}$ the total number of edges between communities. The probability that a specific half-edge will be paired with another specific half-edge equals $1/(2L^{(b)}-1)$, since the half-edges are paired at random. We denote the number of half-edges adjacent to vertex i by $d_i^{(b)}$. Then the contribution of the inter-community edges can be written as

$$\begin{aligned}\mathbb{E}\Big[\frac{1}{N}\sum_{\{i,j\}\in E_b} d_i d_j\Big] &= \frac{1}{N}\sum_{i=1}^{N}\sum_{j=1}^{N}\sum_{k=1}^{d_i^{(b)}}\sum_{l=1}^{d_j^{(b)}}\frac{d_i d_j}{2L^{(b)}-1} = \frac{1}{N}\sum_{i=1}^{N}\sum_{j=1}^{N}\frac{d_i d_i^{(b)} d_j d_j^{(b)}}{2L^{(b)}-1} \\ &\approx \frac{1}{N2L^{(b)}}\left(\sum_{i=1}^{N} d_i d_i^{(b)}\right)^2 = \frac{1}{2L^{(b)}/N}\left(\frac{1}{N}\sum_{i=1}^{N} d_i d_i^{(b)}\right)^2 = \frac{\mathbb{E}[D_N D_N^{(b)}]^2}{\mathbb{E}[D_N^{(b)}]}. \end{aligned} \tag{14}$$

Combining (11), (13) and (14) gives for the expected assortativity of a HCM network that

$$\mathbb{E}[r(G)] = \frac{2\frac{\mathbb{E}[D_N D_N^{(b)}]^2}{\mathbb{E}[D_N^{(b)}]} + 2\frac{\mathbb{E}[Q_n]}{\mathbb{E}[S_n]} - \frac{\mathbb{E}[D_N^2]^2}{\mathbb{E}[D_N]}}{\mathbb{E}[D_N^3] - \frac{\mathbb{E}[D_N^2]^2}{\mathbb{E}[D_N]}}. \tag{15}$$

Supplementary Table 1 shows that HCM and HCM* generate networks that match the assortativity of the original network closer than a configuration model. However, the assortativity generated by HCM does not always match its theoretical value. An explanation for this is that HCM generates simple graphs, while the theoretical estimate does not take this into account. Since both ends of a self-loop have the same degree, having non-simple graphs increases the assortativity. Furthermore, these self-loops typically occur at nodes of large degree, increasing the assortativity even further, so that the theoretical assortativity is higher than the observed assortativity.

3.2 Overlap of HCM and original communities

By keeping the sets of communities fixed, we expect both HCM and HCM* to generate networks with a similar community structure as the original data set. To test how similar the community structures of the generated networks and the original networks are, we define the similarity w_{HCM} of the two community structures as

$$w_{\mathrm{HCM}} = \frac{1}{N}\sum_{i}\frac{|\mathcal{C}_i \cap \mathcal{C}_i^{\mathrm{HCM}}|}{|\mathcal{C}_i|}, \tag{16}$$

where $\mathcal{C}_i$ and $\mathcal{C}_i^{\mathrm{HCM}}$ are the sets of vertices that are in the same community as vertex i in the original network, and the HCM network respectively. We define the similarity for the community structure generated by HCM* similarly as w_{HCM^*}. Table 3 presents this similarity measure for all networks. We see that for most networks, the degree of overlap is large, but for the AS network, the overlap between the original network community sets and the networks generated by HCM or HCM* is smaller. This may be explained by the fact that the AS network has less dense communities, so that rewiring the edges between communities can easily shift vertices from one community to the other.

3.3 Connectedness of HCM* communities

The communities of HCM* are generated by rewiring the edges of the original communities. This may cause the communities to be disconnected after rewiring. Table 3 presents the fraction of disconnected communities f_{dis} that HCM* generates. Table 3 also presents N_{dis}, the average number

of vertices that are not connected to the largest component of the community after rewiring, given that the community is disconnected. We see that the fraction of disconnected communities is different for the different networks. For the networks with a more dense community structure, the probability that a community becomes disconnected after rewiring is low, while for for example the AS network this probability is higher. In all cases, the number of vertices that are disconnected from the largest component is low, indicating that the community stays largely connected.

Supplementary Note 4 Types of epidemic processes

In Figure **??** and Supplementary Figures 1- 5, the results of several epidemic processes are plotted. Here we describe these processes in more detail.

4.1 Bond percolation

In bond percolation, every edge of the network is deleted independently with probability $1-p$. The quantity of interest is the fraction of vertices that are in the largest component after this deletion process.

4.2 Site percolation

In site percolation, every vertex, and all edges adjacent to it, are deleted with probability $1-p$, independently for every vertex. As in bond percolation, we are interested in the fraction of vertices in the largest component after this deletion process.

4.3 Targeted attack

In a targeted attack, a fraction of p of the vertices and the edges adjacent to them are removed, starting with the highest degree vertex, then the second highest degree vertex and so on. Again, the quantity of interest is the fraction of vertices in the giant component after deleting the edges.

4.4 Bootstrap percolation

In bootstrap percolation with threshold t initially a certain fraction of vertices is infective. The initially infected vertices are selected at random. Then, every vertex with at least t infected neighbors also becomes infected. This process continues until no new vertices become infected anymore. In the results, we consider bootstrap percolation with threshold $t = 2$. The quantity of interest is the fraction of infected vertices when the process has stopped.

4.5 SIR epidemic

In an SIR epidemic, vertices are either susceptible, infected or recovered. One vertex is selected uniformly at random to be the initial infective. Then, every infected vertex infects his susceptible neighbors independently at rate β. Every infected vertex recovers at rate γ. As in [27], we set $\gamma = 1$ and $\beta = 3\langle d\rangle/\gamma$, where $\langle d\rangle$ is the average degree of the network. We are interested in how the fraction of infected and recovered vertices evolves over time. Note that since every vertex is either susceptible, infected or recovered, the fraction of susceptible vertices is then also known.

Supplementary Discussion

Supplementary Table 2 shows the fraction of edges of the data sets that are inside communities. HCM fixes all these edges, so one could argue that HCM overfits the data by keeping this fraction of edges fixed. For this reason, we also consider HCM*. Supplementary Figure 6 shows the fraction of rewired edges in HCM* in communities of size s. This is the fraction of edges that are different from

the edges in the original community after the rewiring procedure inside communities. In general, a large fraction of edges is different after randomizing the intra-community edges. The cases where only a few edges were rewired correspond to small communities, where only a small amount of simple random graphs with the same degree distribution exist, or larger communities that are complete graphs, or star-shaped (where only one simple graph with that degree distribution exists). This shows that HCM* creates substantially different graphs than HCM, and is less prone to overfitting the data than HCM.

Supplementary Tables

	**data**	**HCM**	**HCM***	**CM**	**HCM (theory)**
**AS**	-0.19	-0.16	-0.16	-0.14	0.00
**Enron**	-0.11	-0.06	-0.05	-0.05	-0.02
**HEP**	0.27	0.25	0.23	0.00	0.25
**PGP**	0.24	0.26	0.26	-0.01	0.26
**FB**	0.18	0.11	0.10	0.00	0.11
**yeast**	-0.10	-0.03	0.00	-0.01	-0.02

Supplementary Table 1: Assortativity of HCM, HCM* and CM compared to the real network and theoretical HCM value. The theoretical value is derived in Supplementary Note 3.1. The values of HCM, HCM* and CM are averages over 500 generated graphs.

**AS**	**Enron**	**Hep**	**PGP**	**FB**	**yeast**
0.58	0.58	0.70	0.83	0.54	0.52

Supplementary Table 2: The fraction of edges inside communities in the data sets.

	f_{dis}	N_{dis}	w_{HCM}	w_{HCM^*}
**AS**	0.24	3.00	0.68	0.65
**Enron**	0.02	3.62	0.94	0.92
**HEP**	0.04	2.23	0.96	0.94
**PGP**	0.17	2.54	0.97	0.91
**FB**	0.17	2.61	0.93	0.92
**yeast**	0.11	2.29	0.85	0.81

Supplementary Table 3: Connectedness of HCM* communities and overlap of community structure of generated networks and original networks as defined in (16).

Supplementary Figures

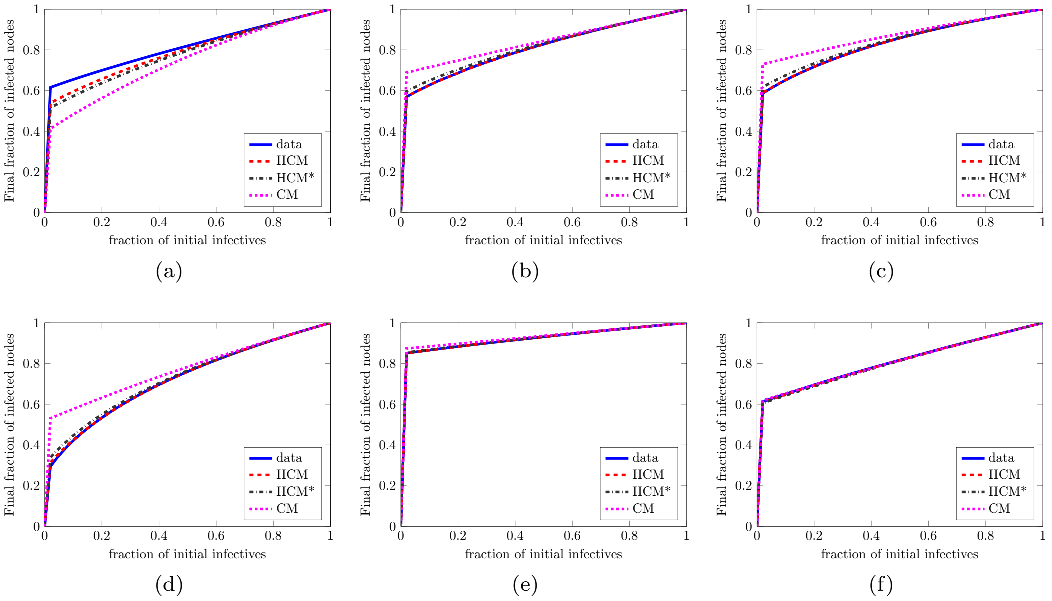

Supplementary Figure 1: **HCM, HCM* and CM under bootstrap percolation compared to real-world networks.** a) Autonomous Systems network b) Enron email network c) Collaboration network in High energy physics d) PGP network e) Facebook friendship network f) yeast network. Initially, a certain fraction of the vertices is infected at random. Then, a vertex becomes infected when at least 2 of its neighbors are infected. The final fraction of infected vertices is the average of 500 generated graphs.

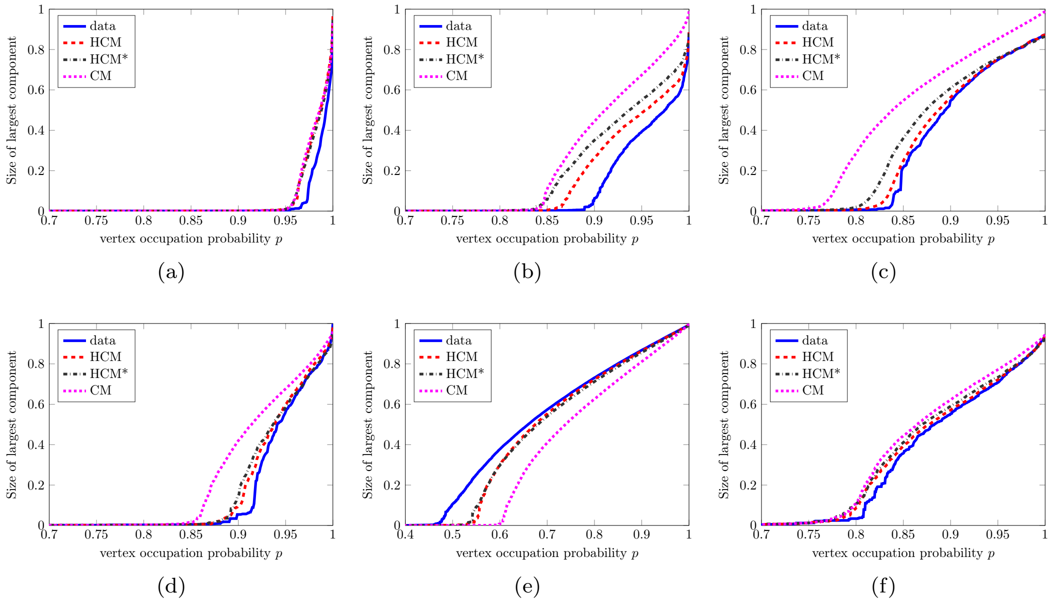

Supplementary Figure 2: **HCM, HCM* and CM under a targeted attack, compared to real-world networks.** a) Autonomous Systems network b) Enron email network c) Collaboration network in High energy physics d) PGP network e) Facebook friendship network f) yeast network. The fraction of $1 - p$ vertices of highest degree are removed. The size of the largest component after the vertices are removed is the average of 500 generated graphs.

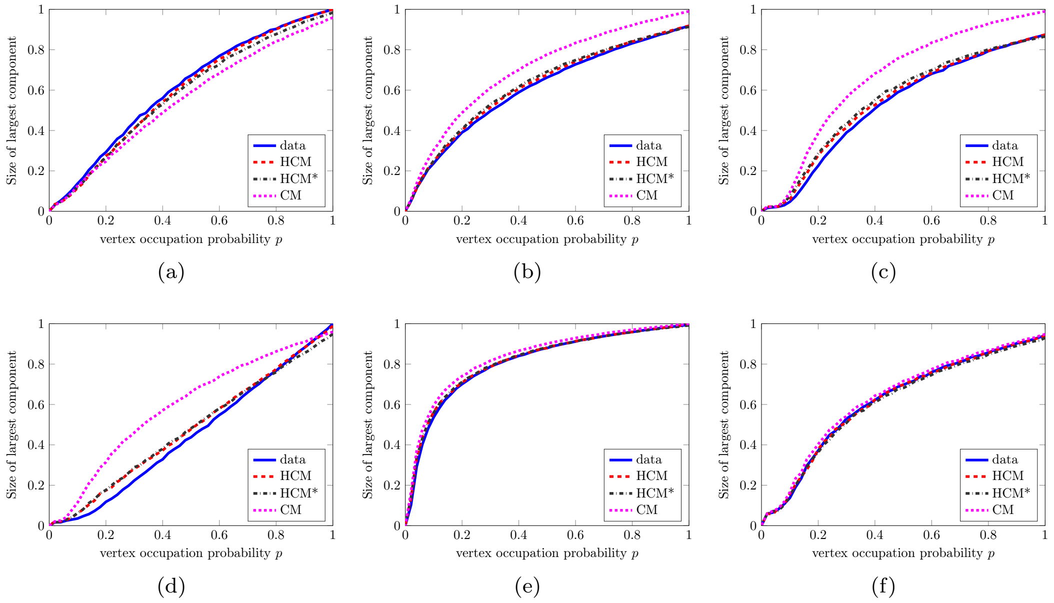

Supplementary Figure 3: **HCM, HCM* and CM under site percolation compared to real-world networks.** a) Autonomous Systems network b) Enron email network c) Collaboration network in High energy physics d) PGP network e) Facebook friendship network f) yeast network. Independently, every vertex is removed from the network with probability $1 - p$. The size of the largest component after the vertices are removed is the average of 500 generated graphs.

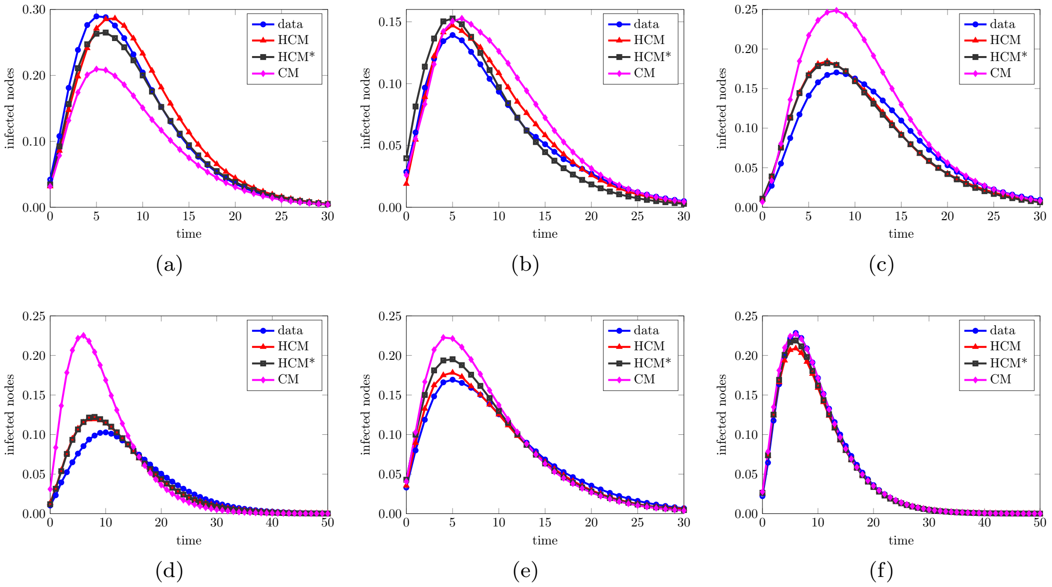

Supplementary Figure 4: **The number of infected individuals in an SIR epidemic in HCM, HCM* and CM compared to real-world networks.** a) Autonomous Systems network b) Enron email network c) Collaboration network in High energy physics d) PGP network e) Facebook friendship network f) yeast network. The presented results are the average of 500 generated graphs, with recovery rate $\gamma = 1$ and infection rate $\beta = 3\langle d \rangle / \gamma$, where $\langle d \rangle$ is the mean degree.

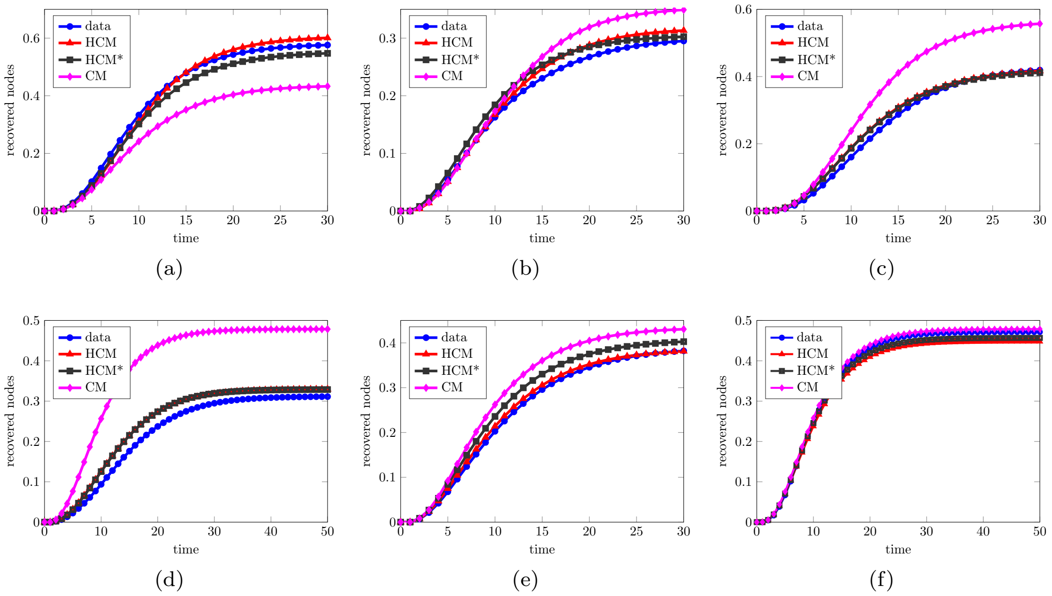

Supplementary Figure 5: **The number of recovered individuals in an SIR epidemic in HCM, HCM* and CM compared to real-world networks.** a) Autonomous Systems network b) Enron email network c) Collaboration network in High energy physics d) PGP network e) Facebook friendship network f) yeast network. The presented results are the average of 500 generated graphs, with the recovery rate $\gamma = 1$ and the infection rate $\beta = 3\langle d \rangle / \gamma$, where $\langle d \rangle$ is the mean degree.

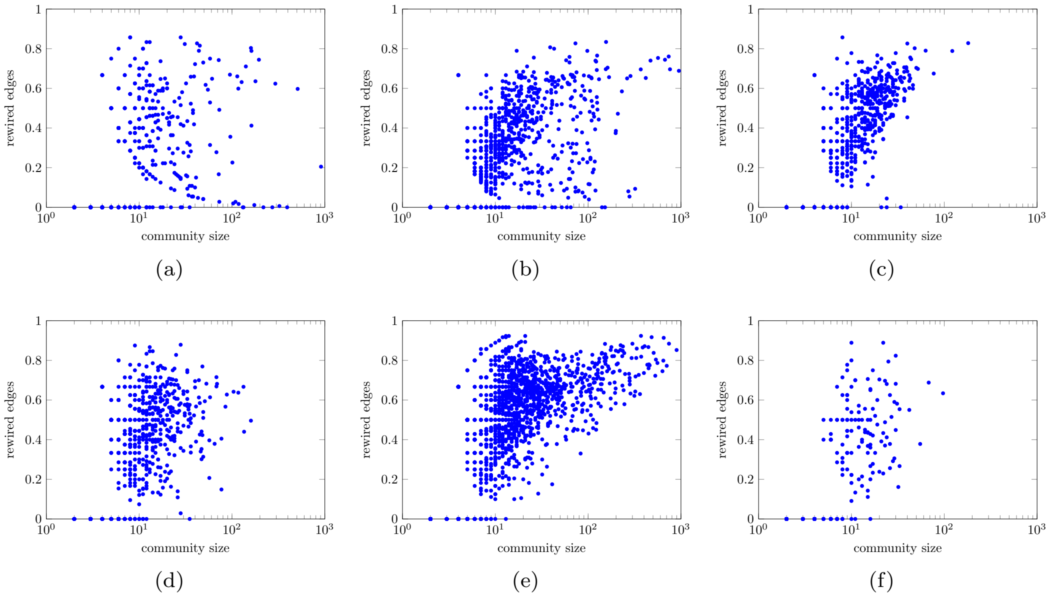

Supplementary Figure 6: **The fraction of rewired edges inside communities for HCM*.** a) Autonomous Systems network b) Enron email network c) Collaboration network in High energy physics d) PGP network e) Facebook friendship network f) yeast network. Every dot corresponds to a community. The fraction of rewired edges is the fraction of edges in the community that are present after randomizing the intra-community edges, but were not present before randomizing.

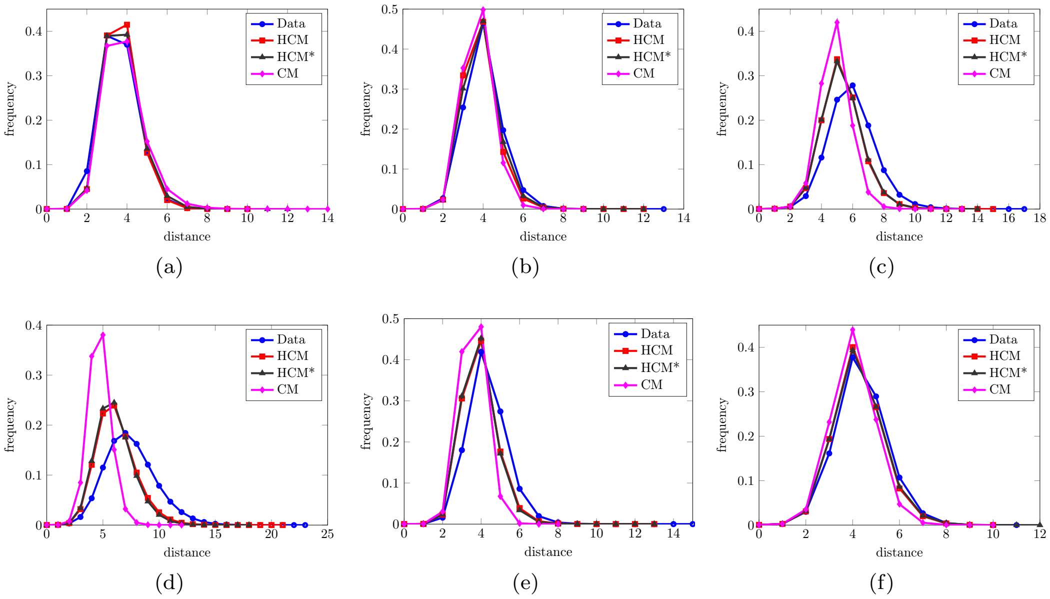

Supplementary Figure 7: **Distances in the original network, HCM, HCM* and CM.** a) Autonomous Systems network b) Enron email network c) Collaboration network in High energy physics d) PGP network e) Facebook friendship network f) yeast network. Distances are approximated by sampling 5,000 nodes from the graphs, and calculating all distances between pairs of nodes in the sampled set. The values for HCM, HCM* and CM are the average over 100 generated graphs.

References

[49] Newman, M. E. J. & Girvan, M. Finding and evaluating community structure in networks. *Phys. Rev. E* **69**, 026113 (2004).

[50] Newman, M. E. J. Mixing patterns in networks. *Phys. Rev. E* **67**, 026126 (2003).

[51] Litvak, N. & van der Hofstad, R. Uncovering disassortativity in large scale-free networks. *Phys. Rev. E* **87**, 022801 (2013).

[52] Lancichinetti, A., Fortunato, S. & Radicchi, F. Benchmark graphs for testing community detection algorithms. *Phys. Rev. E* **78**, 046110 (2008).

[53] Milo, R., Kashtan, N., Itzkovitz, S., Newman, M. E. J. & Alon, U. On the uniform generation of random graphs with prescribed degree sequences. *arXiv preprint cond-mat/0312028* (2003).
